# AngIV-Analog Dihexa Rescues Cognitive Impairment and Recovers Memory in the APP/PS1 Mouse via the PI3K/AKT Signaling Pathway

**DOI:** 10.3390/brainsci11111487

**Published:** 2021-11-11

**Authors:** Xiaojin Sun, Yang Deng, Xinxin Fu, Siyu Wang, Rui Duan, Yingdong Zhang

**Affiliations:** 1 School of Basic Medicine and Clinical Pharmacy, China Pharmaceutical University, Nanjing 211198, China; 3119090247@stu.cpu.edu.cn (X.S.); 3121090295@stu.cpu.edu.cn (Y.D.); 3120090287@stu.cpu.edu.cn (X.F.); 2 Department of Neurology, Nanjing First Hospital, Nanjing Medical University, Nanjing 210006, China; wangsiyu12345@126.com

**Keywords:** Alzheimer’s disease, Dihexa, cognitive, PI3K/AKT

## Abstract

The renin-angiotensin system (RAS) is a paracrine RAS within the central nervous system (CNS) and is closely related to Alzheimer’s disease (AD). The endogenous hexapeptide angiotensin IV (Ang IV), an important component of the brain RAS, was found to rescue cognitive impairment and recover memory in previous studies. In our study, we used different doses of Dihexa, which can be orally administered and cross the BBB in APP/PS1 mice. We found that the amount of AngIV in mouse tissue increased after the administration of Dihexa compared to that in the WT group. Meanwhile, Dihexa restored spatial learning and cognitive functions in the Morris water maze test. Dihexa increased the neuronal cells and the expression of SYP protein in APP/PS1 mice in Nissl staining. Furthermore, Dihexa decreased the activation of astrocytes and microglia, markedly reduced levels of the pro-inflammatory cytokines IL-1β and TNF-α and increased the levels of the anti-inflammatory cytokine IL-10. Dihexa activated the PI3K/AKT signaling pathway, while PI3K inhibitor wortmannin significantly reversed the anti-inflammatory and anti-apoptotic effects of APP/PS1 mice. These findings highlight the brain AngIV/PI3K/AKT axis as a potential target for the treatment of AD.

## 1. Introduction

Alzheimer’s disease (AD) is a central nervous system (CNS) disease that affects individuals aged 65 years old and over. Its incidence varies with age and promotion [1]. It is characterized by the overproduction of extracellular senile plaques composed of the β-amyloid peptide (Aβ), the aggregation of hyperphosphorylated tau protein, and the selective loss of neurons in neuropathology [2,3]. The main manifestations of AD are cognitive impairment, memory loss, language, and behavior disorder, thereby harming the physical and mental health of patients. However, the pathogenesis of AD remains unclear, and medical treatments have a limited effect [4].

The renin-angiotensin system (RAS) is one of the most important enzyme-neuropeptide systems containing ACE, angiotensin II, and AT1R [5,6]. These members are indispensable for the regulation of fluid homeostasis and blood pressure. In recent decades, many studies have established the presence of a paracrine RAS within the CNS. The paracrine RAS consists of the same components and acts independently of peripheral function [7]. AngIV, an important component of the brain RAS, has been linked to neuroprotective effects, such as the improvement of cognitive impairment and recovery of memory in some animal models [8,9,10]. Previous studies have shown that AngIV and its analogs could enhance cognitive ability in rodents and rescue cognitive impairment induced by cerebral ischemia [11,12]. However, the mechanisms are yet to be elucidated. In the pretest, we found that the levels of AngIV were reduced in the brains of APP/PS1 mice. Based on these findings, we hypothesized that the level of AngIV may influence the progression of AD. However, AngIV and most of its analogs have a short half-life and cannot cross the blood–brain barrier (BBB), limiting their use in animal models. Moreover, there are no studies on its effects in an APP/PS1 mouse model. Dihexa (N-hexanoic-Tyr-Ile-(6)-amino hexanoic amide) is an oral active, blood–brain barrier-permeable angiotensin IV analogue. It has a long cyclic half-life and exhibits excellent antidementia activity in scopolamine and aged rat models and marked synaptogenic activity [13,14].

The phosphoinositide 3-kinase (PI3K) class of intracellular enzymes is involved in multiple cell signaling pathways, including the well-studied PI3K-Akt pathway, and is involved in various cellular functions, such as cell growth, proliferation, and differentiation [15]. Furthermore, the PI3K signaling pathway is putatively altered in the development of AD. For example, Cui et al. revealed that the inhibition of PTEN via the activation of the PI3K/AKT pathway attenuated endoplasmic reticulum stress and apoptosis in APP/PS1 transgenic AD model mice [16]. The study showed that Ang IV has a cardioprotective effect against I/R injury by inhibiting apoptosis via the PI3K-Akt-mTOR pathway.

Based on the above, in this study, we aimed to investigate whether Dihexa improves the cognition of APP/PS1 mice and whether the brain AngIV/PI3K/AKT axis is a possible mechanism.

## 2. Materials and Methods

### 2.1. Drugs and Reagents

Dihexa and wortmannin were purchased from MedChemExpress (Monmouth Junction, NJ, USA). AngIVI enzyme-linked immunosorbent assay (ELISA) kits were purchased from JianglaiBiology (Shanghai, China). L-10, TNF-α and IL-1β enzyme-linked immunosorbent assay (ELISA) kits were purchased from RayBiotech (Norcross, GA, USA). Primary antibodies against synaptophysin, GFAP, PI3K, p-AKT, and AKT, were purchased from CST (Danvers, MA, USA). Primary antibodies against Iba-1 and β-actin were purchased from Abcam (Cambridge, UK).

### 2.2. Animals and Treatment

Male (APP/PS1) mice and wild-type (C57) mice were purchased from Beijing Zhishan Company (Beijing, China). Mice were housed in a standard animal room under a 12/12 h day/night cycle and provided free access to food and water. Six-month-old APP/PS1 mice and age-matched WT mice were used in this study. The experiment was divided into two parts: Part 1, in which they were randomly divided into four groups: the WT group, the APP/PS1 group, the APP/PS1+ Dihexa (1.44 mg/kg) group, and the APP/PS1+ Dihexa (2.88 mg/kg) group, and Part 2, in which mice were divided into three groups: the APP/PS1 group, the Dihexa (2.88 mg/kg) group, and the Dihexa (2.88 mg/kg) + wortmannin (0.5 mg/kg) group. The doses of Dihexa and Wortmannin were selected based on a previous study [14,17,18,19]. Dihexa and wortmannin were dissolved in 10% DMSO, 40% PEG300, 5% Tween-80, and 45% saline, and administered intragastrically to the APP/PS1 mice. The Dihexa was administered intraperitoneally to the APP/PS1 mice from six to nine months old. Additionally, 0.9% saline was administered to the WT group once daily for three months. The use of experimental animals in this project conforms to the requirements of experimental animal welfare and ethics, and the procedures involving animals were in accordance with the ethical standards of Nanjing First Hospital (Protocol#: DWSY-1901438).

### 2.3. Morris Water Maze (MWM)

After administering the drug for three months, mice underwent the Morris water maze. We used a round black tub (diameter: 136 cm; depth: 60 cm) filled with water at 18–20 °C. A small hidden platform (10 cm × 6.5 cm × 21.5 cm) was placed in the center of the northeast quadrant of the tank and submerged 1 cm below the water surface. The water maze tank was divided into four equal regions using two mutually perpendicular lines, labeled north (N), south (S), east (E), and west (W). The pool area was conceptually divided into four quadrants of the same size. Each mouse was subjected to four trials per day for five consecutive days. Each mouse was given 60 s to search for the platform. At the end of each trial, each mouse was placed on the platform and allowed to stay there for 30 s. On the sixth day, we removed the platform and recorded the times that the mouse crossed the quadrant in which the platform was located in 60 s [19]. An infrared camera was mounted at the center above the circular pool to record the tracks of the mice. All data were recorded using the EthoVision XT 10.5 system.

### 2.4. ELISA

Following the MWM test, the mice were euthanized by cervical dislocation. The brain tissue was accurately weighed, and PBS was added in the ratio of weight (g) to volume (mL) = 1:9. After homogenization, the supernatant was centrifuged and measured with a sensitive and specific ELISA according to the manufacturer’s instructions for TNF-α, IL-10 and IL-1β (RayBiotech, Norcross, GA, USA). The level of AngIV was measured with a sensitive and specific ELISA according to the manufacturer’s instructions (Jianglai Biology, Shanghai, China).

### 2.5. Nissl Staining

After perfusion, the brain was fixed for 48 h, dehydrated, and embedded in wax. The paraffin-embedded sections were dehydrated with xylene, absolute ethanol, 95% alcohol, 80% alcohol, 70% alcohol, and distilled water. Coronal slices at 4 μm thick were set for the Nissl staining trial. Subsequently, the sections were stained with methylene blue for 15 min. After washing with double distilled water, the sections were dehydrated with 70% alcohol, 80% alcohol, and 95% alcohol for 2 min. Next, the sections were washed twice with anhydrous ethanol for 5 min per wash, and then washed twice with xylene for 5 min per wash. Then, cell morphological alteration of the cortex was observed by microscopy [20]. Compared to normal neurons, the cell bodies of injured neurons were shrunken and/or contained vacuoles and the nuclei stained darker. Positively stained cells were counted using the Image J program. Five random ROIs were selected for quantification, and the mean was used for the statistical analysis. The “% Nissl positive neurons” was calculated by positive neurons/total neurons cells. Each field was imaged at ×100 magnification.

### 2.6. Western Blot

Western blotting was carried out as described previously [21,22], and brain homogenates were lysed on ice for 30 min in 100 mL of lysis buffer (120 mM NaCl, 40 mM Tris (pH 8), and 0.1% NP40) and centrifuged at 12,000 rpm for 30 min. Protein concentrations were determined using a bicinchoninic acid (BCA) assay. A total of 30 μg of protein was separated using 10% SDS-PAGE and electroblotted onto polyvinylidene difluoride (PVDF) membranes using a semi-dry blotting apparatus. After blocking in 5% non-fat milk, the membranes were incubated overnight at 4 °C with primary antibodies. The membranes were then incubated with secondary antibodies for 2 h at room temperature on a shaker. The bands were visualized using Western Lightning ECL Pro with horseradish peroxidase (HRP). β-actin was used as the loading control.

### 2.7. Statistical Analysis

Data were analyzed using GraphPad Prism 8 (GraphPad Software, San Diego, CA, USA). Differences in the escape latency in the Morris water maze test were analyzed using two-way repeated-measures ANOVA. The Student’s *t*-test was used for two group comparisons. The other data were analyzed by one-way ANOVA followed by Tukey’s post hoc test for multiple comparisons. All data are presented as the mean ± SD. *p* < 0.05 was considered significant for all tests.

## 3. Results

### 3.1. Dihexa Restored the Decrease in AngIV in APP/PS1 Mice

**Figure 1:**
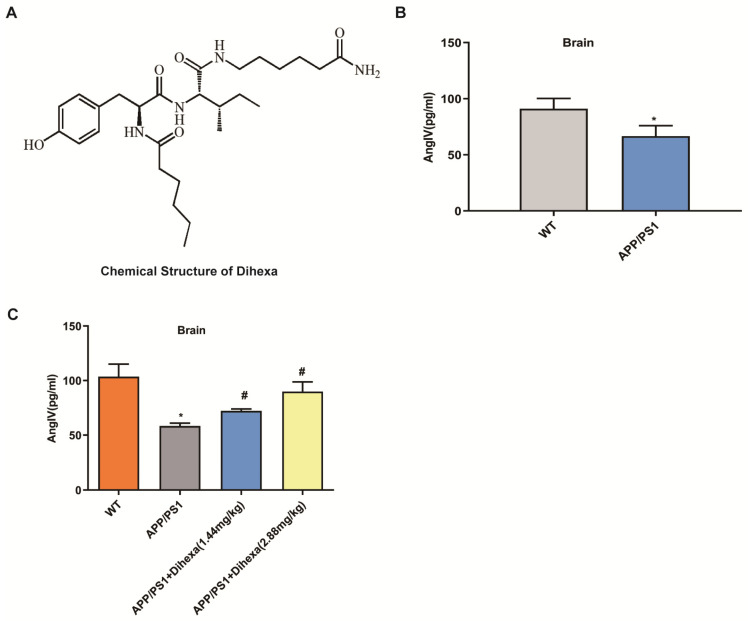
Dihexa restored the decrease in AngIV in APP/PS1 mice. (**A**) The chemical structure of Dihexa. (**B**) AngIV levels in the brains of APP/PS1 and WT mice as detected by ELISA. Student’s *t*-test was used for two group comparisons. Columns represent mean ± SD (*n* = 6 per group) * *p* < 0.05 vs. age-matched vehicle-treated WT control mice. (**C**) Dihexa increases the AngIV levels in the brains of APP/PS1 mice. The data were analyzed by one-way ANOVA followed by Tukey’s post hoc test. Columns represent mean ± SD (*n* = 12 per group). * *p* < 0.05 vs. age-matched vehicle-treated WT control mice. # *p* < 0.05 vs. vehicle-treated APP/PS1 mice.

Figure 1A shows the chemical structure of Dihexa. To identify the AngIV that may be involved in the AD, we first detected the level of AngIV in WT and APP/PS1 mice. As shown in Figure 1B, the level of AngIV in the brains of the APP/PS1 mice was remarkably lower than that of the WT. To validate the findings, we examined the level of AngIV after 1.44 mg/kg and 2.88 mg/kg Dihexa were administered intragastrically to the APP/PS1 group. We were surprised to find that the level of AngIV increased after three months (Figure 1C). In conclusion, AngIV may play a role in the development of AD.

### 3.2. Dihexa Rescued the Cognitive Ability of APP/PS1 Mice

**Figure 2:**
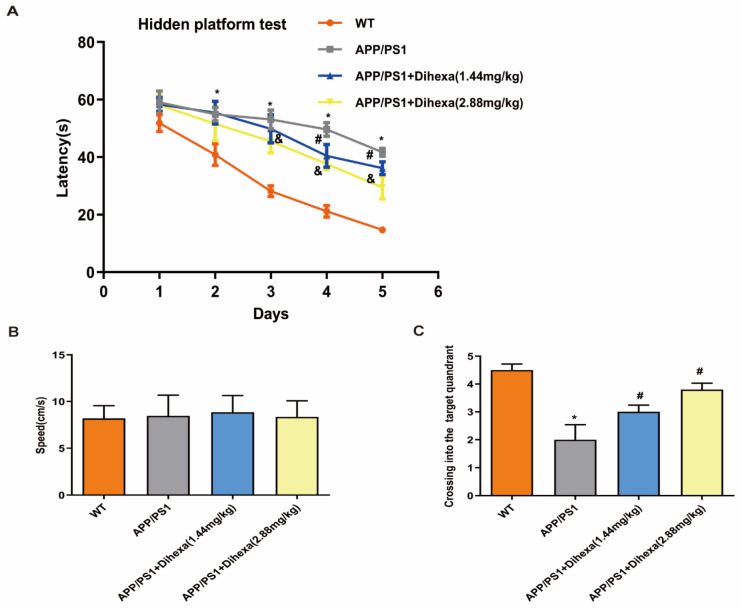
Dihexa rescued the cognitive ability of APP/PS1 mice. (**A**) Dihexa improved spatial learning when locating the hidden platform. Data were analyzed by two-way repeated-measures ANOVA followed by Bonferroni’s multiple comparisons test. (**B**) Swimming speed was not significantly different among the groups during acquisition training. Data were analyzed by one-way ANOVA followed by Tukey’s post hoc test. (**C**) Dihexa tended to increase the number of crossings in the target quadrant. Data were analyzed by one-way ANOVA followed by Tukey’s post hoc test. Columns represent mean ± SD (*n* = 12 per group). * *p* < 0.05 vs. age-matched vehicle-treated WT control mice, # *p* < 0.05, vs. vehicle-treated APP/PS1 mice. ^&^ *p* < 0.05 vehicle-treated APP/PS1 mice.

To investigate the function of Dihexa in APP/PS1 mice, we used MWM to measure the cognitive ability of APP/PS1 mice. As shown in Figure 2A, the escape latency in the four groups decreased from Day 1 to Day 5. However, the escape latency of the APP/PS1 group was significantly higher than that of the WT group. Meanwhile, 1.44 mg/kg and 2.88 mg/kg Dihexa decreased the escape latency to varying degrees, especially on the fourth and fifth days. In the acquisition training, swimming speed did not differ among the groups (Figure 2B). During the probe trial, Dihexa-treated APP/PS1 mice performed significantly better than APP/PS1 controls for the number of platform crossings (Figure 2C). These findings indicate that Dihexa rescued the cognitive ability of APP/PS1 mice.

### 3.3. Dihexa Ameliorated Neuronal Loss in the Brains of APP/PS1 Mice

**Figure 3:**
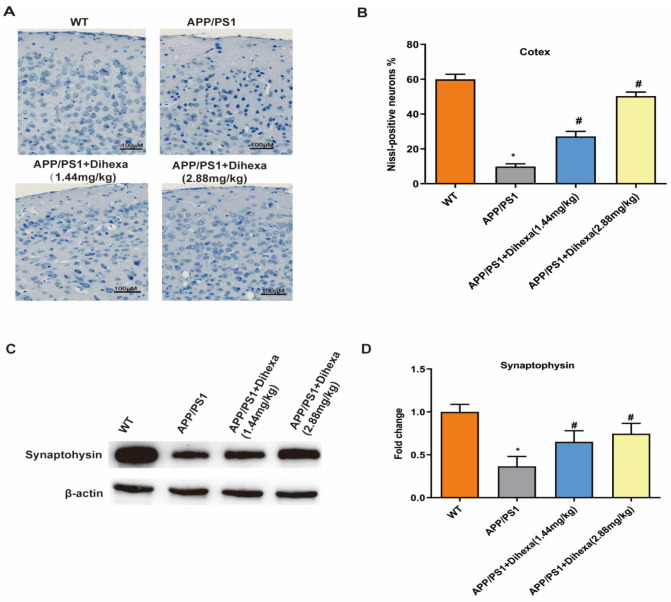
Dihexa ameliorated neuronal loss in the brains of APP/PS1 mice. (**A**) Nissl staining results of the effects of Dihexa on the neuronal cells in the cerebral cortex. Neurons with dark violet nucleus and intact morphology were identified as Nissl-positive neurons. Scale bar = 100μm. (**B**) Quantification analysis of Nissl-positive neuron percentage in cerebral cortex. (**C**) Expression of synaptophysin in mouse brains. (**D**) Densitometric analysis of synaptophysin protein expression. All data were analyzed by one-way ANOVA followed by Tukey’s post hoc test. Columns represent mean ± SD (*n* = 6 per group). * *p* < 0.05 vs. age-matched vehicle-treated WT control mice. ^#^ *p* < 0.05 vs. vehicle-treated APP/PS1 mice.

To determine whether Dihexa mediates neuronal loss, we used Nissl staining to observe the positive neuronal cells. As shown in Figure 3A, APP/PS1 mice exhibited remarkable synaptic loss compared to WT control mice. In addition, the number of neuronal cells decreased in the cerebral cortex of APP/PS1 mice compared to that of WT control mice (Figure 3B). However, the number of neuronal cells significantly increased in Dihexa-treated (1.44 mg/kg and 2.88 mg/kg) APP/PS1 mice. Synaptophysin (SYP) is a synaptic alveolar glycoprotein expressed in neuroendocrine cells. As shown in Figure 3C,D, the expression of SYP protein in the APP/PS1 group was lower than that in the WT group. In addition, the Dihexa-treated APP/PS1 mice had a significantly increased expression of SYP (Figure 3D). Based on these findings, Dihexa ameliorated neuronal loss in the brains of APP/PS1 mice.

### 3.4. Dihexa Attenuated Neuroinflammation and Inhibited Glial Activation in the Brains of APP/PS1 Mice

**Figure 4:**
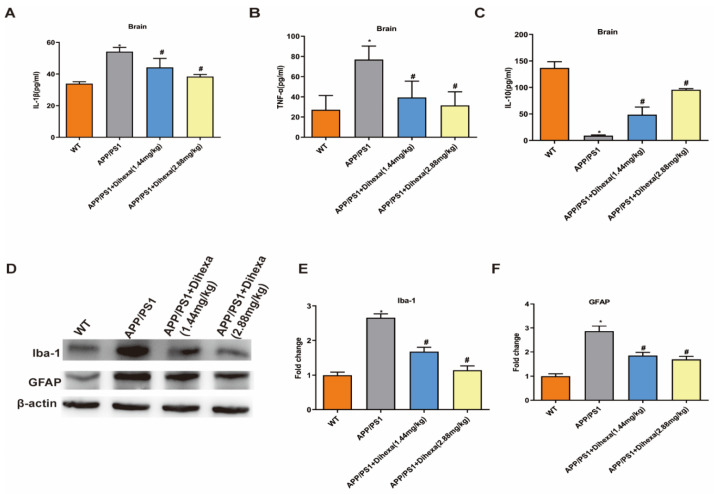
Dihexa attenuated neuroinflammation and inhibited glial activation in the brains of APP/PS1 mice. (**A**–**C**) The levels of IL-1β, TNF-α, and IL-10 in the brain were investigated by ELISA. All data were analyzed by one-way ANOVA followed by Tukey’s post hoc test. (**D**–**F**). The expression of GFAP and Iba-1 and densitometric analysis of the two proteins. Columns represent mean ± SD (*n* = 6 per group). * *p* < 0.05 vs. vehicle-treated WT control mice. # *p* < 0.05 vs. vehicle-treated APP/PS1 mice.

In order to explore the relationship between neuronal apoptosis and inflammatory factors, we detected the levels of IL-1β, TNF-α and IL-10 in this study. Results showed that the levels of TNF-α and IL-1β in the APP/PS1 group were significantly increased compared with those in the WT group (Figure 4A,B). However, Dihexa-treated APP/PS1 mice had significantly decreased levels of TNF-α and IL-1β. As shown in Figure 4C, IL-10 levels in the APP/PS1 group were significantly decreased compared with those in the WT group, and Dihexa-treated APP/PS1 mice had significantly increased levels of IL-10 (Figure 4C). As is known, anti-GFAP is an astrocyte marker and anti-Iba-1 is a microglia marker. As indicated in Figure 4D, the expression of GFAP and Iba-1 in the brains of APP/PS1 mice was significantly upregulated compared with that in WT mice. However, Dihexa-treated APP/PS1 mice had significantly downregulated expression of GFAP and Iba-1. These results showed that Dihexa has a good protective effect on the damage of nerve cells in the brain caused by inflammatory factors.

### 3.5. Dihexa Activated the PI3K/AKT Signaling Pathway in the Brains of APP/PS1 Mice

**Figure 5:**
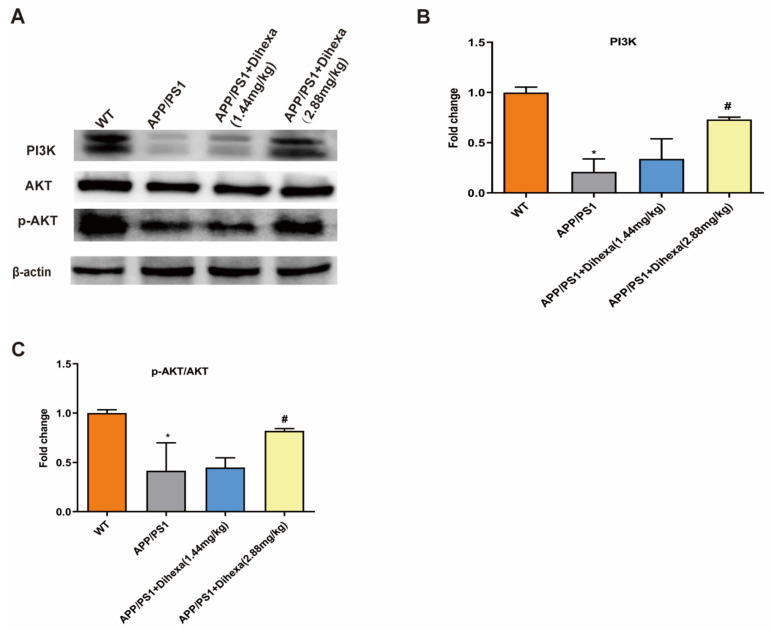
Dihexa activated the PI3K/AKT signaling pathway in the brains of APP/PS1 mice. (**A**) Expression of PI3K, AKT, and p-AKT proteins. (**B**,**C**) Densitometric analysis of PI3K and p-AKT protein expression. All data were analyzed by one-way ANOVA followed by Tukey’s post hoc test. Columns represent mean ± SD (*n* = 6 per group). * *p* < 0.05, vs. age-matched vehicle-treated WT control mice. # *p* < 0.05 vs. vehicle-treated APP/PS1 mice.

To clarify the mechanism of action of the Dihexa, we detected the expression of PI3K/AKT signaling pathway proteins. Compared with that in WT control mice, the ratio of PI3K/β-actin and p-AKT/AKT in the brain was significantly reduced in APP/PS1 mice. Dihexa increased the expression of PI3K and p-AKT (Figure 5A–C). These results indicated that Dihexa activated the PI3K/AKT signaling pathway.

### 3.6. PI3K Inhibitor Reversed the Effect of Dihexa in the Brains of APP/PS1 Mice

**Figure 6:**
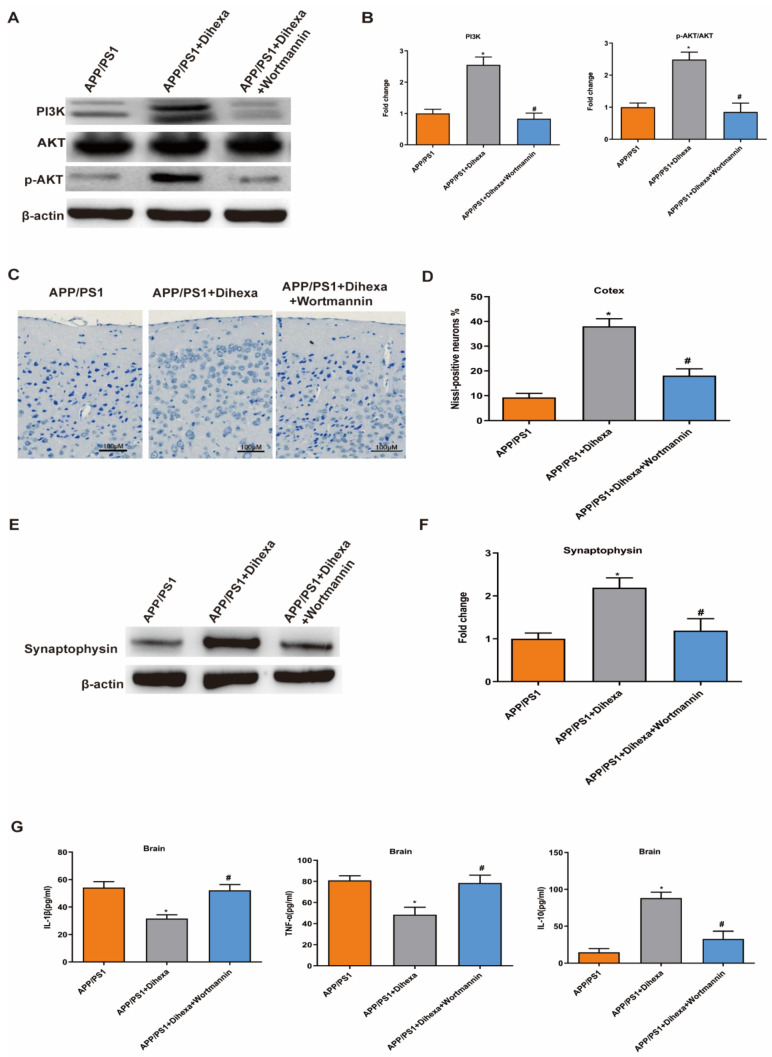
PI3K inhibitor reversed the effect of Dihexa in the brains of APP/PS1 mice. (**A**) Expression of PI3K, AKT, and p-AKT proteins used PI3K inhibitor. (**B**) Densitometric analysis of PI3K and p-AKT protein expression. (**C**) Nissl staining results of the effects of PI3K inhibitor on the neuronal cells in the brain. (**D**) Quantification analysis of Nissl-positive neuron percentage in cerebral cortex. (**E**,**F**) The expression and quantification analysis of SYP by PI3K inhibitor. (**G**) The levels of IL-1β, TNF-α, and IL-10 investigated by ELISA. All data were analyzed by one-way ANOVA followed by Tukey’s post hoc test. Columns represent mean ± SD (*n* = 6 per group). * *p* < 0.05 vs. age-matched APP/PS1mice. # *p* < 0.05 vs. Dihexa-treated APP/PS1 mice.

To validate whether the PI3K/AKT signaling pathway is involved in the mechanism of the Dihexa, we detected the number of neuronal cells and the level of SYP and inflammatory factors in the brains of mice after intragastric administration of wortmannin (a PI3K inhibitor). Results showed that wortmannin significantly reversed the expression of PI3K and AKT (Figure 6A–B). The anti-inflammatory and anti-apoptotic effects of Dihexa were reversed by wortmannin. We found that PI3K inhibition decreased the number of neuronal cells and the expression of SYP (Figure 6C–F). Meanwhile, it increased the levels of TNF-α and IL-1β and decreased the level of IL-10 (Figure 6G). These results indicated that the anti-inflammatory and anti-apoptotic effects of Dihexa are related to the PI3K/AKT signaling pathway.

## 4. Discussion

Alzheimer’s disease, a progressive neurodegenerative disease, is one of the leading causes of death and disability among the elderly, and a heavy burden on families and society [23]. However, only five drugs have been approved for the treatment of AD, but these drugs do not significantly control symptoms or change the course of the disease [24]. With the increasing prevalence of AD and the relative inadequacy of existing medication-based treatments, the development and implementation of new therapeutic drugs is urgently needed. Some studies have indicated that AngIV is involved in the development of AD, and reestablishing its levels in the brain could rescue cognitive impairment and recover memory in some animal models [11,12,25]. In our study, the level of AngIV was reduced in the brains of APP/PS1 mice; this result is consistent with the previous study in APP mice [8]. This indicated that restoring the levels of AngIV may have a positive effect on the progression of AD.

Predictably, results showed that different doses of Dihexa-treated APP/PS1 mice increased the levels of AngIV. The MWM is an important and the main method for measuring spatial learning and memory [26]. To evaluate the effect of Dihexa on cognition and spatial memory, MWM tests were conducted after three months of injection in the APP/PS1 group. APP/PS1 mice spent more time finding the platform under water in the acquisition trial. In the probe trial, the times of crossing the platform in the APP/PS1 group were less than those in the 1.44 mg/kg and 2.88 mg/kg groups. Dihexa significantly decreased the time required to find the platform under the water in the acquisition trial and increased the number of times the platform was crossed. These results indicate a protective effect of Dihexa in rescuing cognitive impairment and recovering memory. This is consistent with the results of previous studies [8,25].

As is known, the most important feature of AD pathology is neuronal loss. The number of neurons in the entorhinal cortex and hippocampus is significantly reduced in patients with AD [24,27]. Nissl bodies can reflect surviving neurons, and these neurons may dissolve and disappear under pathological conditions. Cognitive impairment is associated with synaptic abnormalities in the brain [28]. Synaptic proteins such as synaptophysin (SYN) play an important role in synaptic plasticity and cognitive function. SYN protein levels are lower in elderly individuals with dementia [29]. Research has shown that Dihexa exhibits excellent antidementia activity in scopolamine and aged rat models and is a marker of synaptogenic activity [14]. Therefore, we believe that the increasing number of cortical neurons in the brain and the expression of synaptophysin are important in Alzheimer’s disease. A reduction in neuronal cells is associated with the induction of significant extracellular amyloidosis and neuroinflammatory responses [30]. In addition, in the 9-month-old APP/PS1 transgenic mice, more than half of the hippocampal neurons were lost. Dihexa increases the number of neurons in the brains of APP/PS1 mice. Synaptophysin is a presynaptic vesicle-associated protein found in nearly all synapses of the CNS and is an important synaptic marker [31]. Dihexa increased the expression of synaptophysin in the APP/PS1 mouse brains. These results indicate that the improvement of cognition and spatial memory of APP/PS1 mice is related an increase in neurons.

Neuroinflammatory processes are a central feature in the development of degenerative disease. Inflammatory components, such as microglia, astrocytes, complement systems, and cytokines, are linked to neuroinflammation in the CNS. More specifically, changes in inflammatory cytokines in the brains of mice are a key indicator for evaluating the function of nerve cells. Moreover, the activation of glia cells has been reported in both AD patients and animal models, accompanied by an activated complement system and increased levels of chemokines and cytokines [30,32,33,34,35]. IL-1β, a potential pro-inflammatory factor and member of the cytokine interleukin-1 family, is derived from microglia and macrophages. TNF-α is a multipotent pro-inflammatory cytokine secreted by a variety of cells, including adipocytes, activated monocytes, macrophages, B cells, T cells, and fibroblasts [36]. In inflammation, IL-10 can reduce the antigen-presenting function by downregulating the expression of Mononuclear Cell Surface II major histocompatibility antigens (Class II major histocompatibility complex, MHC II), and inhibit inflammatory cell activation, migration, and adhesion by downregulating the activity of T cells. At the same time, IL-10 also inhibits the synthesis and release of inflammatory factors [37]. In the Elisa assay, we found that Dihexa decreased the level of pro-inflammatory factors Il-1β and TNF-α. Furthermore, it increased the level of anti-inflammatory factor IL-10. Thus, Dihexa restores the structure and function of nerve cells related to the change in inflammatory cytokines. As is known, microglia and astrocytes play an important role in regulating the levels of inflammatory cytokines in the brain [38]. Meanwhile, we evaluated the expression of microglia and astrocyte markers in the brains of mice. It was found that the expression of GFAP and Iba-1 was restored significantly following Dihexa administration. In addition, 2.88 mg/kg Dihexa significantly improved the recovery of nerve cells and function in AD mice compared with 1.44 mg/kg Dihexa. It has been shown that Dihexa can restore the structure and function of nerve cells by regulating the inflammatory response and preventing nerve cell damage caused by the overexpression of Aβ, thus reducing the inflammatory response in the brains of AD mice.

The PI3K/AKT signaling pathway is closely related to the mechanisms of cell survival, cell metabolism, and apoptosis. Current studies have found that this pathway may be involved in the pathogenesis of brain aging, AD, and other diseases [39,40]. In the study of AD, it was found that due to the reduced expression of AKT in hippocampal neurons, the phosphorylation of BAD and CREB was reduced, leading to neuronal apoptosis [41]. In addition, some studies have confirmed that the activity of the PI3K/AKT signaling pathway is also involved in the development of neuronal dendrites and the formation of dendritic spines. Previous studies have shown that the PI3K/AKT signaling pathway plays anti-neuroinflammation, anti-oxidative stress and anti-apoptosis roles in neurons and increases AKT signaling, which inhibits the release of proinflammatory cytokines [42,43,44]. Thus, the PI3K/AKT signaling pathway is related to the occurrence and development of AD to a certain extent, and the pathogenesis of AD can be regulated by changing the role of the PI3K/AKT signaling pathway. In our results, Dihexa increased the expression of PI3K and phosphorylated AKT. Subsequently, PI3K inhibition by the specific inhibitor wortmannin significantly reversed the anti-inflammatory and anti-apoptotic effects of APP/PS1mice. We believe that the effect of improving cognitive recovery memory is exerted through this signaling pathway.

However, it should be noted that this study has some limitations. First, whether doses larger than 2.88 mg/kg would further improve MWM performance is worth exploring in future studies. Second, in the current study, treatment with wortmanin reversed the effects of Dihexa in the APP/PS1 model. However, it would have been more accurate to have added an “only wortmanin” control in Figure 6.

## 5. Conclusions

In conclusion, we suggest that Dihexa rescues cognitive impairment and recovers memory by ameliorating neuronal loss and inhibiting inflammation and glial activation via PI3K/AKT signaling pathways in APP/PS1 mice.

## Data Availability

The data presented in this study are available on request from the corresponding authors.
